# Modulation of Adult Mesenchymal Stem Cells Activity by Toll-Like Receptors: Implications on Therapeutic Potential

**DOI:** 10.1155/2010/865601

**Published:** 2010-06-14

**Authors:** Olga DelaRosa, Eleuterio Lombardo

**Affiliations:** Cellerix S.A, Parque Tecnológico de Madrid, Calle Marconi 1, Tres Cantos, 28760 Madrid, Spain

## Abstract

Mesenchymal stem cells (MSCs) are of special interest as therapeutic agents in the settings of both chronic inflammatory and autoimmune diseases. Toll-like receptors (TLR) ligands have been linked with the perpetuation of inflammation in a number of chronic inflammatory diseases due to the permanent exposure of the immune system to TLR-specific stimuli. Therefore, MSCs employed in therapy can be potentially exposed to TLR ligands, which may modulate MSC therapeutic potential in vivo. Recent results demonstrate that MSCs are activated by TLR ligands leading to modulation of the differentiation, migration, proliferation, survival, and immunosuppression capacities. However inconsistent results among authors have been reported suggesting that the source of MSCs, TLR stimuli employed or culture conditions play a role. Notably, activation by TLR ligands has not been reported to modulate the “immunoprivileged” phenotype of MSCs which is of special relevance regarding the use of allogeneic MSC-based therapies. In this review, we discuss the available data on the modulation of MSCs activity through TLR signalling.

## 1. Introduction

Adult mesenchymal stem cells (MSCs) represent an innovative tool for cell-based therapy of degenerative disorders, chronic inflammatory, and autoimmune diseases or allograft rejection. The understanding of the mechanisms that mediate and/or modulate the therapeutic potency of MSCs is important from both a physiological and a clinical point of view. In this context, one key interest is to better understand the modulation of MSC biology by Toll-like receptors (TLRs) as they have been linked to the perpetuation of chronic inflammatory responses (Chron's disease, Rheumatoid Arthritis) through the recognition of conserved pathogen-derived components or endogenous ligands (also known as “danger signals”) that MSCs will likely encounter in the sites of injury. Here we will review the most recent data on these topics.

## 2. Adult Mesenchymal Stem Cells and Cell Therapy

### 2.1. Differentiation Capacity of MSCs

MSCs have been isolated from multiple tissues of mesodermal origin, such as bone marrow (BM-MSCs), adipose tissue (AD-MSCs), umbilical cord blood and peripheral blood. MSCs can be easily isolated by adhesion to plastic and expanded *in vitro* in serum containing media with no additional requirements for growth factors or cytokines. In culture, they acquire a fibroblast-like morphology. Despite ample efforts, no exclusive surface markers have been identified for MSCs. Thus, to date they are defined according to the criteria of the International Society for Cellular Therapy by being negative for hematopoetic and endothelial markers such as CD11b, CD14, CD31, CD34, and CD45, and positive for a variety of many other markers, including HLA class I, CD105, CD73, CD29, and CD90 [1]. Therefore, MSCs can be identified *in vitro* by their ability to differentiate into mesenchymal-type cells (adipocytes, osteoblasts, and chondrocytes) but also neurons, endothelial cells, astrocytes, and epithelial cells when cultured in the appropriate conditions [2–6].

### 2.2. Immunogenicity of MSCs

The expression of human leukocyte antigen (HLA) molecules class I (also called Mayor Histo-Compatibility class I = MHC I) on all cells on the body allows the immune system to distinguish self from nonself. In the absence of immune suppression or tolerogenic mechanisms, allogeneic cells are rejected by the immune system upon recognition of their foreign HLA. Cells expressing HLA molecules stimulate T cells directly only if they possess appropriate costimulatory molecules—CD80 (B7-1), CD86 (B7-2) or CD40-. Allogeneic cells can also activate T cells through an indirect pathway where their HLA antigens are presented by professional antigen presenting cells (APC). Aside from HLA class I, certain cells also express HLA class II constitutively or after induction. HLA II also plays an important role in antigen presentation and immune response. A remarkable unique feature of MSCs is that they are considered to be immunoprivileged as they express low levels of cell-surface HLA class I molecules whereas HLA class II, CD40, CD80, and CD86 are not detectable on the cell surface. Stimulation with interferon (IFN)-*γ* has been shown to increase both class I and class II molecules, however, MSCs do not express costimulatory molecules CD80 (B7-1), CD86 (B7-2) or CD40, even after IFN-*γ* stimulation. These features allow MSCs to escape to the immune surveillance [7–11]. MSCs not only fail to induce activation of CD4+ cells but also escape lysis by CD8+ cytotoxic lymphocytes [12]. Even whole lymphocytes stimulated *in vitro* to target Peripheral Blood Lymphocytes (PBLs) derived from a specific donor, will lyse lymphocytes from that individual but not MSCs derived from the same donor. Further analyses indicate that MSCs induce an abortive activation programme in fully differentiated CD8+ T cells so that major effector functions are not activated. MSCs also escape natural killer (NK) cell-specific lysis [12].

### 2.3. Immunosuppressive Capacity of MSCs

 A third important feature of MSCs is that they are immunosuppressive and inhibit activation, proliferation, and function of immune cells, including T cells, B cells, NK cells and antigen-presenting cells (APCs) [6, 13–24]. Despite ample research in recent years, mostly in BM-MSCs, the specific molecular and cellular mechanisms involved in the immunoregulatory activity of MSCs remain controversial. There is evidence that the capability to modulate immune responses relies on both cell contact-dependent mechanisms and soluble factors secreted by MSCs in response to cytokines released by activated immune cells. MSCs may inhibit lymphocyte proliferation by a mechanism that requires, at least in part, the release of soluble factors such as hepatocyte growth factor (HGF), prostanglandin-E2 (PGE2), transforming growth factor (TGF)-*β*1, indoleamine 2,3-dioxygenase (IDO), nitric oxide, and interleukin (IL)-10 [14, 16, 18, 21, 22, 25–29]. On the other hand, other studies have shown that BM-MSCs may modulate T-cell phenotype resulting in the generation of cells with regulatory activity [14, 15, 19, 30–34].

### 2.4. MSCs Treatment as a New Therapeutic Tool

The biological characteristics mentioned above make MSCs an interesting tool for cellular therapy and regeneration. This is supported by a number of studies in animal models of inflammatory diseases demonstrating an efficient protection against allograft rejection, graft-versus-host disease, experimental autoimmune encephalomyelitis, collagen-induced arthritis, sepsis and autoimmune myocarditis [13, 28, 31–38]. In fact, MSCs are being used in several clinical trials with a focus on their immunomodulatory capacities (http://clinicaltrials.gov/search/term=stem+cells?term=stem+cells). In this regard, Cellerix is currently conducting a phase III clinical trial using human AD-MSCs to treat complex peri-anal fistula in Crohn patients after having successfully completed phase I and II trial with high efficacy rates [39, 40]. Importantly, it is believed that the therapeutic potency, safety, and efficacy of treatment of inflammatory diseases with MSCs reside to a large extent in their immunologically privileged phenotype and in their immunosuppressive capacity. Interestingly, TLR activation has been implicated in the pathology of a number of inflammatory diseases including rheumatoid arthritis or inflammatory bowel disease (IBD), since they can either initiate or perpetuate the chronic inflammation due to the continue exposure to TLR ligands [41, 42]. Therefore, the use of MSCs in cell therapy for the treatment of inflammatory diseases deserves further investigation regarding the potential effects of TLR signaling on MSCs biology and the potential implications in the immunogenicity and immunosuppressive capacity, which are of special relevance in terms of therapeutic potency.

## 3. Modulation of MSCs through TLRs

### 3.1. MSCs Express Active TLRs

Expression of TLRs at the RNA and protein levels have been studied by RT-PCR, flow cytometry, and immunofluorescence. It is well established that MSCs express a number of TLRs. So far, consistent results demonstrate high mRNA expression of TLR 1, 2, 3, 4, 5, and 6 in AD-MSCs and BM-MSCs from both human and mice, while inconsistent results have been reported on the expression of TLR 7 to 10 in the same studies. At the protein level, expression of TLR2, TLR3, TLR4, TLR7, and TLR9 has been reported by flow cytometry and immunofluorescence [23, 28–31, 43]. 

Expression of TLRs can be modulated in MSCs. Thus, Hwa Cho et al. have studied whether hypoxia can affect the expression of TLRs in human AD-MSCs,as these cells may be used as a therapeutic approach in ischemic tissues [44]. They demonstrated that exposure to hypoxic conditions significantly increase mRNA of TLR1, 2, 5, 9, and 10. Moreover, lipopolysaccharide (LPS) challenge downregulates TLR2 and TLR4 expression in MSC-derived osteoprogenitors [45]. On the other hand, transduction of MSCs with baculoviruses (a DNA viral vector) upregulates expression of TLR3 and triggers TLR3 signaling pathway [46].

**Figure 1:**
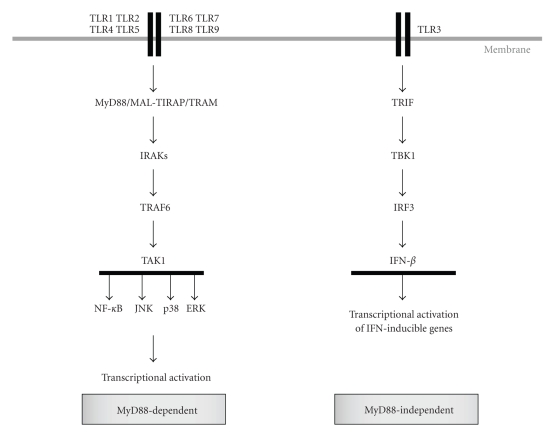
TLR signalling. Ligand recognition results in the recruitment of intracellular TIR-domain-containing adaptors proteins, including myeloid-differentiation primary-response protein 88 (MyD88, shared by all TLRs, except TLR3), and Toll/IL-1R domain-containing adaptor-inducing IFN-*β* (TRIF, employed by TLR3 and TLR4). Engagement of MyD88 activates a signaling cascade including IL-1R-associated kinases (IRAKs,), (TNF)-receptor-associated factor 6 (TRAF6) and transforming growth factor-*β* (TGF-*β*)-activated kinase (TAK1), leading to the activation of the mitogen-activated protein (MAP) kinases ERK, JNK, and p38, and nuclear translocation of the transcription factor nuclear factor-*κ*B (NF-*κ*B) (MyD88-dependent pathway). There is a second, alternative pathway triggered by TRIF (MyD88-independent pathway) that culminates in the activation of NF-*κ*B, MAPKs, and the transcription factors interferon-responsive factors (IRFs), whose are responsible for induction of type I IFNs, in particular IFN*β*. Besides MyD88 and TRIF, two other adaptor proteins have been described: TIR-domain-containing adaptor protein (TIRAP, also called MAL), and TRIF-related adaptor molecule (TRAM, required for TRIF-dependent signaling through TLR4, but not TLR3).

As reviewed extensively somewhere else in this issue, TLR activation trigger MyD88 dependent and independent downstream signalling cascades leading to the nuclear translocation of NF-*κ*B and other transcription factors and the activation of a number of genes (see Figure 1) [47–52]. It has been demonstrated that when AD-MSCs or BM-MSCs were stimulated with ligands specific for different TLRs the nuclear factor-kappa B (NF-*κ*B), mitogen-activated protein (MAP) kinases (MAPKs), PI3K signalling pathways were activated with a subsequent induction of several genes and cytokines, mainly CXCL-10, IL-6 and IL-8. These results clearly demonstrate that MSC express active and functional TLRs. However, differences in the induction of genes in response to TLR activation have been reported. For instance, in contrast to our observations in AD-MSC, Hwa Cho et al., and Tomchuck et al. have reported the induction of tumor necrosis factor (TNF)-*α* and IL-1*β* by LPS and polyinosinic:polycytidylic acid (Poly IC) in BM-MSC and AD-MSC, respectively [44, 53].

### 3.2. Effect of TLR Activation on MSC Survival

In response to TLR stimulation human AD-MSCs induce the expression of manganese superoxide dismutase (MnSOD), a key protective protein against oxidative stress in the mitochondria [23]. It has been reported that induction of MnSOD protects cells from oxidative stress leading to increased survival [54]. In the settings of an inflammatory response, immune cells release vast amounts of reactive oxygen species which results in the generation of an oxidative milieu. Based on these data we speculated that increased expression of MnSOD by MSCs in response to TLR ligand exposure would provide them with improved engraftment or survival at injured or inflamed sites, leading to enhanced therapeutic effects [23]. This hypothesis is further supported by recent results showing that TLR4 activation protects MSCs from oxidative stress-induced apoptosis [55]. In fact, LPS preconditioning of mouse BM-MSCs can, when compared to unconditioned MSCs, improve their survival and engraftment of MSCs and increase the release of vascular endothelial growth factor (VEGF) in a model of rat acute myocardial infarction leading to an enhanced therapeutic effect (improved cardiac function, reduced apoptosis of myocardium, reduced fibrosis and elevated vascular density after myocardial infarction) [56] (see Table 1).

### 3.3. Effect of TLR Triggering in the Differentiation of MSCs

As indicated above, one of the main features of MSCs is the potential to differentiate to several cell types of mesenchymal origin. Some groups have reported the effects of TLR activation on MSC differentiation with contradictory results. We found no effect on adipogenic differentiation but detected that Poly I : C and LPS increased osteogenic differentiation in human AD-MSCs [23]. However Hwa Cho et al. [44] reported increased osteogenic differentiation of human AD-MSC by LPS and peptidoglycan (PGN) activation, whereas CpG oligodeoxynucleotides (CpG-ODN) impaired it. The increased osteogenic differentiation in the presence of LPS or PGN was accompanied by increased ERK activation. These authors reported reduced adipogenic differentiation when PGN was present. On the other hand, whereas Mo et al. [45] reported increased osteogenic differentiation of human BM-MSCs after prolonged LPS activation, Liotta et al. [57] found no effect of TLR activation on adipogenic, osteogenic or condrogenic differentiation of human BM-MSCs. However, Pevsner-Fischer et al. [58] reported that TLR2 activation by Pam3Cys reduced mouse BM-MSC differentiation into the three mesodermal lineages. 

Interestingly, they also found that myeloid-differentiation primary-response protein 88 (MyD88)-deficient BM-MSCs, when cultured in the appropriate differentiation media without additional stimulation with TLR ligands effectively differentiated into adipocytes but failed to differentiate into osteocytes and chondrocytes, indicating that this pathway may be involved in MSC mutipotency. These discrepancies could be due to differences in culture conditions, between bone marrow and adipose-derived MSCs, and between mouse and human cells (see Table 1).

### 3.4. Effect of TLR Activation on Proliferation and Migration of MSCs

The effect of TLR activation in the proliferation of MSCs has been studied by several groups. Stimulation with LPS or Pam3Cys promoted proliferation of mouse BM-MSCs [55, 58], but stimulation with LPS, Poly I : C, lipoteichoic acid (LTA) and PGN showed not significant effects on human AD-MSCs and BM-MSCs [23, 44, 45]. However, Hwa Cho et al. reported that stimulation of AD-MSCs with CpG-ODNs leads to a G1 arrest wich results in inhibition of proliferation [44] (see Table 1).

Migration to the appropriate site of injury is considered to play an important role in the therapeutic efficacy of MSCs. In this context, Tomchuck et al. demonstrated that TLR3 activation drives the migration of human BM-MSCs using transwell and Boyden chamber migration assays suggesting that this TLR signalling pathway may be manipulated to increase the biodistribution of infused MSCs at the injured sites [53]. Moreover, LPS, ODNs, LL-37 (an antimicrobial peptide), fibronectin fragment III 1C (Fn III1C) and flagellin resulted in moderate to limited induced migration. On the other hand, Pevsner-Fischer et al. found that TLR2 activation impaired mouse BM-MSC migration using “wound healing” assays [58] (see Table 1). Nevertheless, further investigation will be required to better understand the potential role of TLR signalling in migration and biodistribution of MSCs *in vivo*, which is of great clinical relevance.

### 3.5. TLRs and the Immunogenic Phenotype of MSCs

The potential use of allogeneic MSCs relies on the special capacity of these cells to escape to the immune recognition. It is well established that TLR activation can modulate expression of costimulatory molecules in immune cells. Therefore, it is of special interest from a therapeutic point of view to determine whether exposure of MSCs to TLR ligands may induce the expression of HLA-I, HLA-II, and costimulatory molecules (CD40, CD80, CD86) leading to an augmented immunogenic phenotype. We analyzed the expression of HLA-I, HLA-II, CD80, and CD86 in human AD-MSCs by flow cytometry 72 hours after stimulation with LPS, Poly I : C, and PGN. We found that LPS, Poly I : C, and PGN did not alter the expression of HLA-II, CD80, and CD86. Poly I : C was the only TLR ligand capable to induce HLA-I to some extend. Furthermore, costimulation of human AD-MSCs with IFN-*γ* (a well-known inducer of HLA-I and HLA-II expression in MSCs) in combination with either LPS, Poly I : C or PGN did not alter the INF-*γ*-mediated induction of HLA-I and HLA-II [23]. Similar results have also been reported in human BM-MSCs [43, 57]. These results indicate that TLR activation does not significantly affect the immunogenic properties of human MSCs (see Table 1). These results are of great relevance regarding the use of allogeneic MSC-based cell therapies.

### 3.6. Effect of TLR Activation on the Immunosuppressive Capacity of MSCs

BM-MSC and AD-MSCs have been shown to possess the capacity to inhibit proliferation of immune cells upon mitogenic or allogeneic activation. As mentioned above, this immunosuppressive capacity of all MSCs can become a key factor for their therapeutic use and potency. The mechanisms underlying the immunosuppression potential of MSCs are not fully understood, but seem to require both cell-to-cell contact-dependent mechanisms and the release of soluble immune modulators (IDO, PGE2, TGF-*β*1, nitric oxide, etc.) upon activation in response to immune cells. Interestingly, some of these immune modulators are downstream of signalling pathways triggered by TLRs in other cell types. Therefore, a feasible hypothesis is that TLR ligands may induce the production of such anti-inflammatory mediators in MSCs resulting in an enhanced immunosuppressive phenotype. Moreover, TLR signalling has been associated with the perpetuation of chronic inflammatory and autoimmune diseases such as Crohn's disease and Rheumatoid Arthritis [41, 42]. Therefore, AD-MSCs and BM-MSCs employed in the treatment of such diseases will likely be exposed to TLR ligands, which may result in the modulation of MSCs activity and therapeutic potency. Therefore, it is very important to determine whether TLR signalling may modulate the immunosuppressive capacity of MSCs.

In recent years, several groups have reported inconsistent results regarding the role of TLR ligands on the modulation of MSCs capacity to suppress immune responses. In this context, we tested the role of TLRs in the immunosuppressive capacity of human AD-MSCs. To do so, we analyzed proliferation of activated CFSE-labeled PBLs, CD4+ T cells and CD8+ T cells in the absence or presence of increasing amounts of human AD-MSCs precultured for up to 72 hours with medium alone or in the presence of LPS, Poly I : C or PGN. We found no significant effect of TLR activation on human AD-MSC-mediated suppression, indicating that activation through TLR2, TLR3, and TLR4 do not significantly interfere with the capacity of human AD-MSCs to modulate immune responses *in vitro*. Supporting these results, IDO, a key mediator of human AD-MSCs immunosuppression [24] was weakly induced by a high concentration of Poly I : C and was not induce upon TLR2 or TLR4 triggering [23]. Similar results were reported by Pevsner-Fischer et al., showing that TLR2 activation by Pam3Cys does not affect immunosupression mediated by mouse BM-MSC [58]. However, other groups have reported that TLR activation may modulate the immunosuppressive properties of human BM-MSCs, although in very different ways. Thus, Liotta et al. found that TLR3 and TLR4 activation reduce the inhibitory activity of human BM-MSCs on T-cell proliferation without influencing IDO activity or PGE2 levels [57]. By using inhibitors of the Notch signalling pathway and anti-Jagged-1 neutralizing antibodies they found that TLR activation leads to the downregulation of the Notch ligand Jagged-1 in BM-MSCs. Based on these data, the authors suggested that Notch signalling pathway mediates the cell contact-mediated immunosuppression by MSCs. In contrast to these results, Opitz et al. have recently reported that TLR3 and TLR4 engagement enhances the immunosuppressive properties of human BM-MSCs through the indirect induction of IDO1 [43]. Induction of IDO1 involved an autocrine IFN-*β* signalling loop, which was dependent on protein kinase R (PKR) and independent on IFN-*γ*. 

A common characteristic of all MSCs is that they constitutively express IL-6 and IL-8. The significance of a constitutive expression of IL-6 and IL-8, which can both be considered proinflammatory cytokines, in context to the immunosuppressive activity of MSCs is still unclear. Interestingly, it has been reported that MSCs inhibit the differentiation of dendritic cells, at least in part, through the release of IL-6 [59]. This observation links IL-6 production to the immunosuppression mediated by MSCs. Hence, it is tempting to speculate that induction of IL-6 secretion by TLR activation may enhance MSCs-mediated impairement of dendritic cells differentiation and maturation.

These inconsistent and partly contradictory results demonstrate the complexity of the immune system, even under *in vitro* conditions. It is likely that differences in the experimental settings between laboratories might be behind these contradictory results (see Table 1). For instance, the use of PBMCs versus purified T cells or the method of activation of these cells may play a role. Liotta et al. employed purified CD4+ T cells stimulated with allogeneic T cell-depleted PBMCs and anti-CD3 mAb, Opitz et al. used a mixed lymphocyte reaction (MLR) with total PBMC from two unrelated donors, one of which was irradiated, and we used either whole PBMC samples or purified CD4+ and CD8+ fractions stimulated with beads loaded with anti-CD3, anti-CD2 and anti-CD28 mAbs. Another important aspect could be the time of treatment with TLR ligands and the concentration of them (very variable among studies). While some left TLR ligands in the cocultures or pretreated the MSCs with TLR ligands for several days before initiating the coculture experiments [23, 57], others pretreated MSCs for 24 hours, washed and cocultured them with the MLR [43]. It has also been suggested that a long-term exposure to TLR ligands may lead to downregulation of factors induced shortly after activation of TLR [43]. 

As several studies have reported benefitial effects of MSCs treatment in animal models of LPS-induced sepsis or lung injury [32, 38, 60], an inhibition of a therapeutic capacity of MSC by TLR ligands does not appear to be the case. Thus, it is unclear whether *in vivo* potency of MSCs can be potentiated by TLR ligands, however it does not appear to be impaired.

## 4. Concluding Remarks

It is necessary to better define the role of TLR activation on MSC biology in the context of the development of new therapeutic strategies on inflammatory or autoimmune diseases, simply because MSCs are likely be exposed to activation through TLR ligands in the sites of injury or inflammation. The discrepancies shown by different authors need further investigation as it would be relevant to determine whether or not TLR activation interferes or enhances migration, biodistribution or immunosuppressive capacity of MSCs. Differences in the source of MSCs, type and concentration of the stimuli used, the experimental settings and method of detection or culture conditions may explain these discrepancies. On the other hand, the recognition of endogenous ligands by TLRs is now thought to have an important role in the regulation of inflammation, both in infectious and noninfectious diseases. A number of endogenous ligands have been identified, including heat shock protein (HSP) 60, HSP 70, heparan sulfate, hyaluronan, fibronectin extra domain A, uric acid, oxidized LDL, intracellular components of fragmented cells, myeloid-related proteins-8 and 14, eosinophil-derived neurotoxin, and human defensin-3 [61–72]. As these ligands are accessible to TLRs in the setting of injury or non-infectious threat, they have been called “danger signals”. A very important aspect that has not been studied in details so far is the activation and modulation of MSC activity by these danger signals. Given the capacity of MSCs to modulate immune responses it would be interesting to determine whether resident MSCs may be activated by danger signals in the settings of injury and if this activation results in a contribution of MSCs to the process of healing and cure in homeostasis.

## Figures and Tables

**Table 1:** Summary of reported effects on MSC activity by TLR ligands.

Ligand	Adipogenic	Condrogenic	Osteogenic	Immuno-genicity	Immuno-suppression	Migration	Proliferation	Survival	Source	Species	Ref
LPS	≈		↑	≈	≈		≈		AD	Human	[23]
				≈	↑				BM	Human	[43]
	≈		↑				≈		AD	Human	[44]
			↑				≈		BM	Human	[45]
							↑	↑	BM	Mouse	[55]
								↑	BM	Mouse	[56]
						↑			BM	Human	[53]
	≈	≈	≈	≈	↓				BM	Human	[57]

PolyIC	≈		↑	≈	≈		≈		AD	Human	[23]
				≈	↑				BM	Human	[43]
	≈		≈				≈		AD	Human	[44]
						↑			BM	Human	[53]
	≈	≈	≈	≈	↓				BM	Human	[57]

PGN	≈		≈	≈	≈		≈		AD	Human	[23]
	≈		↑				≈		AD	Human	[44]

CpG-ODNs	≈		≈						AD	Human	[23]
	≈		↓				↓		AD	Human	[44]
						↑			BM	Human	[53]

Flagellin	≈		≈						AD	Human	[44]
						↑			BM	Human	[53]

Pam3Cys	↓	↓	↓		≈	↓	↑		BM	Mouse	[58]

LL-37						↑			BM	Human	[53]

Fn III1C						↑			BM	Human	[53]

R-848	≈	≈	≈	≈	≈				BM	Human	[57]

LTA						≈			BM	Human	[45]

≈ (no significant effect), ↑  (increase), ↓  (inhibition), AD (adipose derived MSCs), BM (Bone marrow MSCs). LPS: Lipopolysaccharide; PolyIC: Polyinosinic:polycytidylic acid; PGN: Peptidoglycan; CpG-ODNs: CpG oligodeoxynucleotides; Fn III1C: Fibronectin fragment III 1C; LTA: Lipoteichoic acid.
